# The Global Cognition, Frontal Lobe Dysfunction and Behavior Changes in Chinese Patients with Multiple System Atrophy

**DOI:** 10.1371/journal.pone.0139773

**Published:** 2015-10-02

**Authors:** Bei Cao, Bi Zhao, Qian-Qian Wei, Ke Chen, Jing Yang, RuWei Ou, Ying Wu, Hui-Fang Shang

**Affiliations:** Department of Neurology, West China Hospital, SiChuan University, Chengdu, Sichuan, China; University of Ulm, GERMANY

## Abstract

**Background:**

Studies on cognition in multiple system atrophy (MSA) patients are limited.

**Methods:**

A total of 110 MSA patients were evaluated using Addenbrooke's Cognitive Examination-Revised (ACE-R), Frontal Assessment Battery (FAB), Frontal Behavioral Inventory (FBI), and Unified MSA Rating Scale (UMSARS) tests. Fifty-five age-, sex-, education- and domicile-matched healthy controls were recruited to perform the FAB and ACE-R scales.

**Results:**

Approximately 32.7% of the patients had global cognitive deficits with the most impaired domain being verbal fluency and visuospatial ability (26.4%), followed by memory (24.5%), language (20%) and orientation/attention (20%) based on a cut-off score of ACE-R ≤ 70. A total of 41.6% of the patients had frontal lobe dysfunction, with inhibitory control (60.9%) as the most impaired domain based on a cut-off score of FAB ≤14. Most patients (57.2%) showed moderate frontal behavior changes (FBI score 4–15), with incontinence (64.5%) as the most impaired domain. The binary logistic regression model revealed that an education level < 9 years (OR:13.312, 95% CI:2.931–60.469, P = 0.001) and UMSARS ≥ 40 (OR: 2.444, 95%CI: 1.002–5.962, P< 0.049) were potential determinants of abnormal ACE-R, while MSA-C (OR: 4.326, 95%CI: 1.631–11.477, P = 0.003), an education level < 9 years (OR:2.809 95% CI:1.060–7.444, P = 0.038) and UMSARS ≥ 40 (OR:5.396, 95%CI: 2.103–13.846, P < 0.0001) were potential determinants of abnormal FAB.

**Conclusions:**

Cognitive impairment is common in Chinese MSA patients. MSA-C patients with low education levels and severe motor symptoms are likely to experience frontal lobe dysfunction, while MSA patients with low education levels and severe motor symptoms are likely to experience global cognitive deficits. These findings strongly suggest that cognitive impairment should not be an exclusion criterion for the diagnosis of MSA.

## Introduction

Multiple system atrophy (MSA) is a sporadic adult-onset neurodegenerative disease characterized by any combination of parkinsonism, cerebellar ataxia, autonomic dysfunction and pyramidal tract dysfunction [1]. When parkinsonism is predominant, the disorder is referred to as MSA-P, and when cerebellar ataxia is predominant, this disease is referred to as called MSA-C[1]. Although according to the Diagnostic and Statistical Manual of Mental Disorders dementia is an exclusion criterion for the diagnosis of MSA[2], increasing studies have suggested that cognitive impairment in MSA might be more frequent than previously recognized[3–7]. Furthermore, previous imaging[6–8] and neuropathological studies[9,10] have shown that frontal and temporal lobe degeneration in patients with MSA might contribute to cognitive impairment and behavior changes in MSA.

A recent meta-analysis showed that cognitive impairment is an important and common symptom of MSA[3]. The Addenbrooke’s Cognitive Examination-Revised (ACE-R) was developed as a brief cognitive assessment instrument incorporating elements of the Mini-Mental State Examination (MMSE). This multidimensional test not only facilitates the overall assessment of cognition but also provides an assessment of the profile of different cognitive domains. The ACE-R can be administered without trained personnel and can be completed in approximately 15 minutes. The validation of different ACE-R versions has been conducted in several countries[11–14], and this test has been demonstrated as superior to the MMSE in the detection of cognitive dysfunction in patients with neurodegenerative diseases, such as Parkinson’s disease (PD)[15], MSA[16,17], progressive supranuclear palsy (PSP)[16], corticobasal degeneration (CBD)[16], Alzheimer’s disease (AD)[18], Huntington’s disease (HD)[17] and amyotrophic lateral sclerosis (ALS)[19]. The Chinese version of the ACE-R is a reliable assessment tool for screening cognitive dysfunction in ALS, mild cognitive impairment (MCI) and AD with the different cut-off scores of 75,86 and 68, respectively [13,19].

Frontal-executive dysfunction is the most common presentation of cognitive impairments in MSA patients[3]. The Frontal Assessment Battery (FAB) is a short tool to assess frontal lobe /executive function[20]. A few studies have applied FAB to examine frontal lobe dysfunction in MSA patients[4,21,22], and they showed that 26.7% to 41% of patients with MSA exhibited frontal lobe dysfunction. However, the limitations of these studies included the absence of exploring the relationships between frontal lobe dysfunction and global cognitive status and motor and non-motor symptoms of MSA patients.

Behavior changes have been associated with frontal lobe dysfunction[23]. Because of the evidence of frontal degeneration in MSA patients[6–10], behavior changes in MSA should be considered. The Frontal Behavioral Inventory (FBI), recommended to assess behavior changes[23], has not been applied to study the behavior changes of patients with MSA. Studies focusing on the characteristics of frontal lobe dysfunction and behavioral deficits in Chinese MSA patients are not available. It has been reported that the MSA-C subtype accounts for the majority of MSA in Asian populations, whereas the MSA-P subtype accounts for the majority of MSA in Caucasian populations[24,25]. Thus, whether the characteristics of cognition in Chinese MSA patients are different from those Caucasian populations remains unknown.

Therefore, the following are the aims of the present study: 1) explore the general features of global cognitive status, frontal lobe function and behavior changes in Chinese MSA patients using three screening measurements, i.e., ACE-R, FAB and FBI; 2) reveal the relationship between global cognitive status and frontal lobe function and other important motor and non-motor symptoms of MSA patients; 3) analyze the relationship between behavior changes and other important motor and non-motor symptoms of MSA patients; and 4) reveal the differences in the frontal lobe function and behavior changes between patients of different sexes and subtypes (MSA-P and MSA-C).

## Patients and Methods

A total of 115 MSA patients admitted to the Department of Neurology, West China Hospital, Sichuan University (Tertiary Referral Center of Southwest of China) between April 2012 and April 2014 were enrolled in the present study. All MSA patients met the *probable* MSA clinical diagnostic criteria[2]. All patients were subjected to brain MRI scans to exclude other neurological disorders. Most patients with MSA-C (57/67) were screened for mutations in causative genes of spinal cerebellar ataxia (SCA), including *SCA* 1, 2, 3, 6, and 7 genes, to exclude the diagnosis of SCA. All patients were evaluated during face-to-face interviews. Clinical information regarding sex, age of onset, disease duration, and years of education were collected. The severity of MSA was assessed using the Unified MSA Rating Scale (UMSARS)[26]. The patients were categorized as the MSA-C subtype when cerebellar ataxia symptoms and signs were observed predominately and the MSA-P subtype when parkinsonism symptoms and signs were observed predominated. Patients with severe ataxia (more than 3 points of speech, item 2 of UMSARS-II) or parkinsonism symptoms (more than 3 points of tremor, item 5 of UMSARS-II) were excluded to avoid interfering with cognitive measurements. In addition, patients suffering from a manifest dementia indicated by a Mini-Mental State Examination (MMSE) scores <20 were excluded in accordance to the current MSA consensus criteria[2]. A total of 110 patients with MSA were included in the analysis. The disease onset was defined as the time of initial symptom of any motor (parkinsonism or cerebellar dysfunction) or autonomic feature (except for erectile dysfunction)[1]. The education level was defined as the years of schooling, with reference to the education system in China plus the years after school during which the subject acquired professional qualification. Fifty-five healthy controls (HCs) were matched to 110 patients for age, sex, and education at a ratio of 1:2. HCs were recruited to perform FAB and ACE-R scales measurements. None of the HCs had any neurological diseases and psychiatric disorders.

The global cognition was assessed using ACE-R, which evaluates five cognitive domains including orientation/ attention, memory, verbal fluency, language and visuospatial ability domains, and has a maximal total score of 100, with a higher score representing better cognitive function. Neurologists administered the Chinese versions of both the ACE-R (http://www.ftdrg.org) and MMSE to all patients and HCs by in a standardized manner. Cognitive impairment was defined as a total score of more than 1.5 standard deviations below the mean performance of the mean ACE-R score for the healthy controls (HCs)’ or an MMSE score less than 26[19]. In MSA, there is no specific definition for cognitive impairment and previous studies also measured the cognition of MSA in different ways. We used 1.5 SD as a cut-off score because mild cognitive impairment in Parkinson's Disease (PD-MCI) used 1.5 SD as well [27,28].

The FAB comprised six subtests, including similarities, lexical fluency, motor series, conflicting instructions, go-no-go, and prehension behavior. However, different cut-off scores were selected according to previous studies on MSA. For example, one study used a cut-off score of 10[22], whereas another study used a cut-off score of 14[21]. In the present study, the cut-off score for FAB was defined as a total score of more than 1.5 standard deviations below the mean FAB score for the HCs.

The personality and behavior disturbance was assessed using the 24-item FBI, completed by the caregivers[23]. A value of 0 to 3 was allocated for each item. Total FBI scores ranged from 0 to 72. A total score of zero indicates no behavior changes, a total score of 1 to 3 corresponds to mild behavior changes, a score of 4 to 15 indicates moderate behavior changes, and a total score > 15 corresponds to severe behavior changes[29].

Written informed consent was obtained from all participants prior to recruitment and this study was approved by the Ethics Committee of West China Hospital of Sichuan University.

## Statistical Analysis

Except for FBI-positive score, FBI-negative score and all six domains of FAB, all the continuous data in our study were normally distributed by using the Kolmogorov–Smirnov test. Comparisons of the normally distributed data between groups were analyzed using analysis of variance (ANOVA), while non-normally distributed data were compared by Mann-Whitney U-tests. The normally distributed data were expressed as the mean ± standard deviation (SD), while non-normally distributed data were showed as median (lower quartiles, upper quartiles). The Chi-Square test was used to compare the categorical variables. Spearman’s correlation test was used to assess the relationship between clinical variables, including age of onset, subtype, disease duration, education level, and UMSARS, ACE-R, FAB, and FBI scores. Binary logistic regression (Wald forward entry method) was used to investigate THE multivariate predictors of global cognitive impairment and frontal lobe dysfunction in turn. The examined dichotomized variables included sex (male/female), age (≥60/<60 years), education (≥9/<9 years), disease duration (≥3/<3 years), and UMSARS (≥40/<40) as previously described[21]. Significance was defined as a p value of less than 0.05. The p values were corrected for the false discovery rate to adjust for multiple comparisons. Data analysis was performed using SPSS 21.0 statistical software.

## Results

Among 110 MSA patients including 62 males and 48 females, 67 patients were MSA-C patients and 43 were MSA-P patients. The mean age of the MSA patients was 56.7 ± 9.4 years, and the mean disease duration was 2.7 ± 1.5 years (ranged from 0.55 to 6.7 years). The mean education level was 9.4 ± 3.7 years, and the mean UMSARS score was 38.9 ± 12.5 (ranged from 16 to 76). The 55 HCs included 32 males and 23 females. There were no significant differences in age, sex and education years between the HCs and patients. The demographic data and ACE-R scores for the HCs are presented in Table 1.

**Table 1:** Normative data of healthy controls on the ACE-R and FAB tests.

Variables	Mean (SD)	Range	Cut-off score
Male/female	31/24		
Age (years)	58.4(9.4)	34–76	_
Years of education	8.6(3.9)	1–17	_
ACE-R score	83.2(8.6)	72–98	70
Orientation/attention	17.5(0.9)	14–18	15
Memory	22.2(3.1)	15–26	17
Verbal fluency	10.8(2.3)	6–16	6
Language	19.3(4.0)	12–26	12
Visuospatial ability	13.5(2.1)	8–16	9
FAB score	16.7(1.4)	13–18	14

ACE-R: Addenbrooke's Cognitive Examination-Revised; FAB: Frontal Assessment Battery.

**Fig 1:**
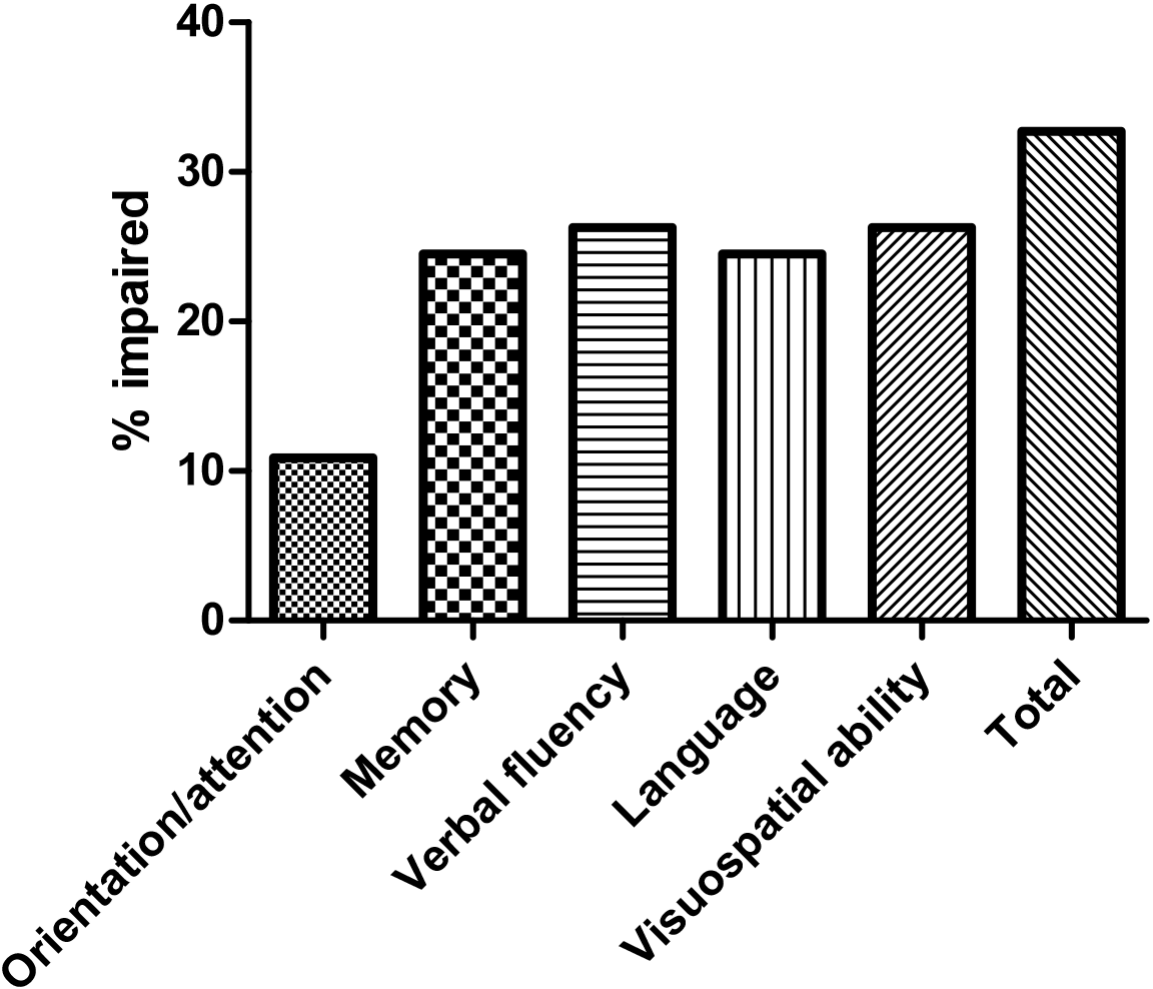
The frequencies of the ACE-R scores for MSA patients less than 1.5 standard deviations below the mean of healthy controls.

In the present study, the cut-off score for ACE-R was set as “70” based on the mean ACE-R score for the HCs (83.2 ± 8.6) (Table 2). The mean ACE-R score for the patients was 73.9 ± 13.4. Therefore, the prevalence of global cognitive deficits based on a cut-off score of ACE-R ≤ 70 was 32.7% (36/110) (Fig 1). The frequency of cognitive deficit of ACE-R domains was 26.3% for verbal fluency, 26.3% for visuospatial ability, 24.5% for language, 24.5% for memory and 10.9% for orientation/attention (Fig 1). The ACE-R-abnormal group had a lower education level, longer disease duration, lower FAB score and higher frequency of abnormal FAB (Table 2). However, based on the MMSE score, 23 patients (20.9%) had cognitive deficits.

**Table 2:** Demographic and clinical characteristics of the MSA patients in terms of global cognition status.

	Global cognition status		
Variables	ACE-R-normal (score>70)	ACE-R-normal (score≤70)	p
Number	64	36	
MSA-C/MSA-P	44/30	23/13	0.665
Men/women	45/29	17/19	0.178
Age of onset (years)	57.0±9.9	56.0±8.3	0.582
Years of education	10.7±3.5	6.9±2.7	<0.0001
Disease duration	2.4±1.4	3.2±1.4	0.013
UMSARS	37.6±13.0	47.6±11.1	0.116
FAB score	15.5±2.3	12.0±3.5	<0.0001
Similarity	3.0(2.0, 3.0)	2.0(1.25, 3.75)	<0.0001
Lexical fluency	3.0(3.0, 3.0)	2.0(2.0, 3.0)	<0.0001
Motor series	3.0(2.0, 3.0)	2.0(1.0, 3.0)	0.038*
Conflicting instruction	3.0(2.0, 3.0)	2.0(1.0, 3.0)	<0.0001
Go-no-go task	2.0(1.0, 3.0)	1.0(0.25, 2.75)	<0.0001
Prehension behavior	3.0(3.0, 3.0)	3.0(3.0, 3.0)	0.006
FAB abnormal	15(20.2%)	23(63.9%)	<0.0001
FBI score	4.9±4.0	6.8±5.3	0.104
FBI positive symptoms	0.0(0.0, 2.0)	0.5(0, 2.0)	0.858
FBI negative symptoms	3.0(1.0, 6.0)	4.5(2.0, 7.75)	0.080
FBI abnormal	59(79.7%)	32(88.9%)	0.233
Mild behavior changes	16(21.6%)	9(25%)	0.665
Moderate behavior changes	42(56.8%)	21(61.8%)	0.875
Severe behavior changes	1(1.4%)	2(5.6%)	0.518

MSA: Multiple system atrophy; FAB: Frontal Assessment Battery; FBI: Frontal Behavioral Inventory; UMSARS: Unified MSA Rating Scale; ACE-R: Addenbrooke's Cognitive Examination-Revised

*after Multiple comparison of the p values adjusted using the false discovery rate approach, which were not significant.

**Fig 2:**
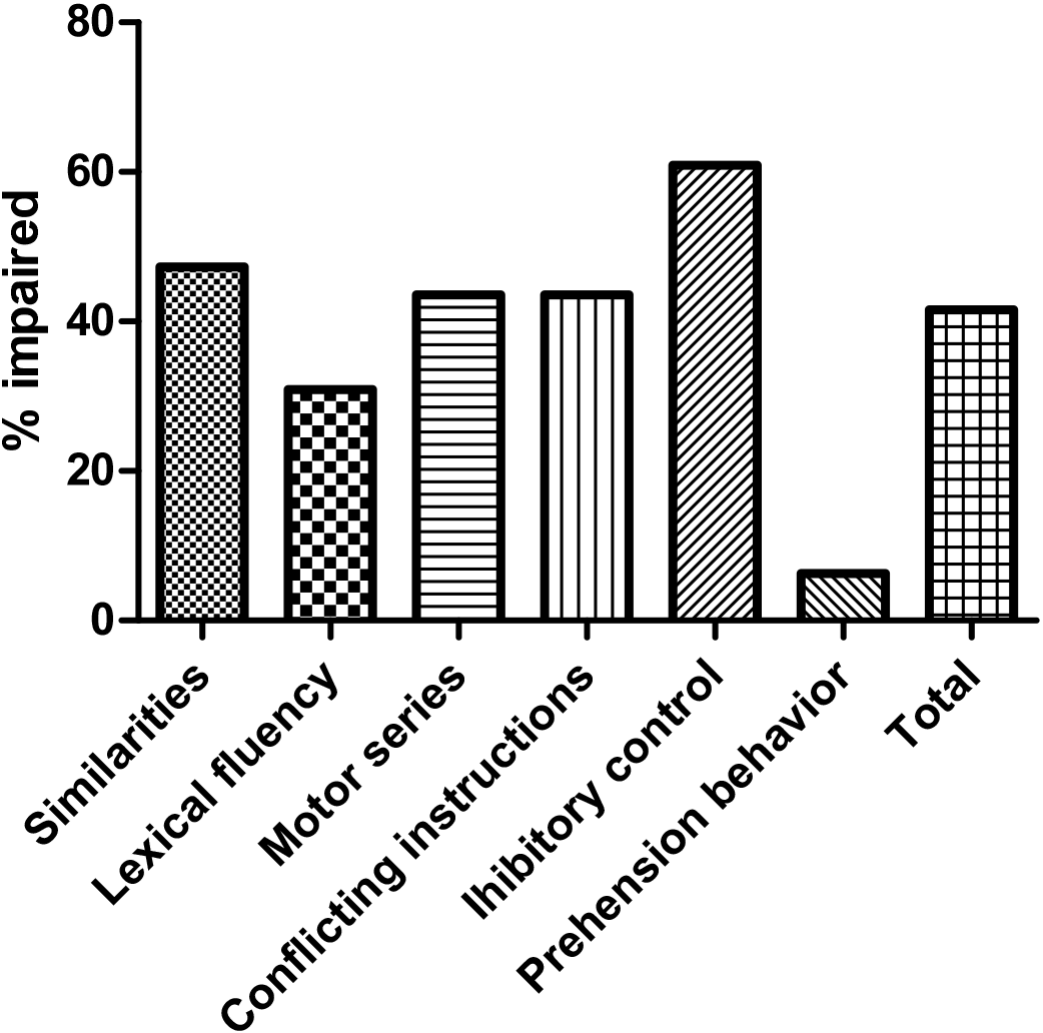
The frequencies of FAB scores less than 3 in each subscale in MSA patients. The maximum score attainable for each subscale is 3.

In the present study, the cut-off score for FAB was set as “14” based on the mean FAB score for the HCs (16.7 ± 1.4) (Table 3). The mean FAB score for the patients was 14.4 ± 3.2. The prevalence of frontal lobe dysfunction was 41.6% (46/110) based on a cut-off score of FAB ≤14 (Fig 2). The most frequent affected subtest was inhibitory control (60.9%) (Fig 2). The FAB-abnormal group had a higher proportion of MSA-C patients with a lower education level, higher UMSARS score, lower ACE-R score and higher frequency of abnormal ACE-R (Table 3). The frequencies of each subtest score of FAB < 3 in MSA patients are presented in Fig 2.

**Table 3:** Demographic and clinical characteristics of the MSA patients in terms of frontal functional status.

	Frontal functional status		
Variables	FAB-normal (score>70)	FAB-normal (score≤70)	p
Number	64	46	
MSA-C/MSA-P	33/31	34/12	0.018
Men/women	36/28	26/20	0.977
Age of onset (years)	55.8±9.6	58.0±9.1	0.231
Years of education	10.4±3.7	8.1±3.3	0.001
Disease duration	2.4±1.2	3.1±1.6	0.018
UMSARS	35.3±11.5	44.0±12.2	<0.0001
ACE-R score	80.0±10.3	65.4±12.7	<0.0001
Orientation/attention score	17.0±1.4	16.0±1.9	<0.0001
Memory score	21.4±3.6	18.3±4.9	<0.0001
Verbal fluency score	8.6±2.3	6.3±3.2	<0.0001
Language score	19.8±4.3	15.0±5.2	<0.0001
Visuospatial ability score	13.2±2.8	9.7±3.4	<0.0001
ACE-R abnormal	12(18.8%)	24(5%)	<0.0001
Orientation/attention abnormal	4(4.1%)	8(28.1%)	0.005
Memory abnormal	9(13.9%)	18(44.7%)	<0.0001
Verbal fluency abnormal	9(15.3%)	20(47.4%)	<0.0001
Language abnormal	6(11.1%)	23(55.3%)	<0.0001
Visuospatial ability abnormal	7(9.7%)	22(57.9%)	<0.0001
FBI score	4.9±4.2	6.4±4.8	0.082
FBI positive symptoms	0.0(0.0, 2.0)	1.0(0.0, 2.25)	0.774
FBI negative symptoms	3.0(1.0, 5.75)	3.5(2.0, 7.25)	0.057
FBI abnormal			
Mild behavior changes	15(23.4%)	10(21.7%)	0.834
Moderate behavior changes	34(53.1%)	29(63.0%)	0.300
Severe behavior changes	1(1.5%)	2(4.3%)	0.570

MSA: Multiple system atrophy; FAB: Frontal Assessment Battery; FBI: Frontal Behavioral Inventory; UMSARS: Unified MSA Rating Scale; ACE-R: Addenbrooke's Cognitive Examination-Revised; after Multiple comparison of the p values adjusted using the false discovery rate approach. All “p” values were significant.

**Fig 3:**
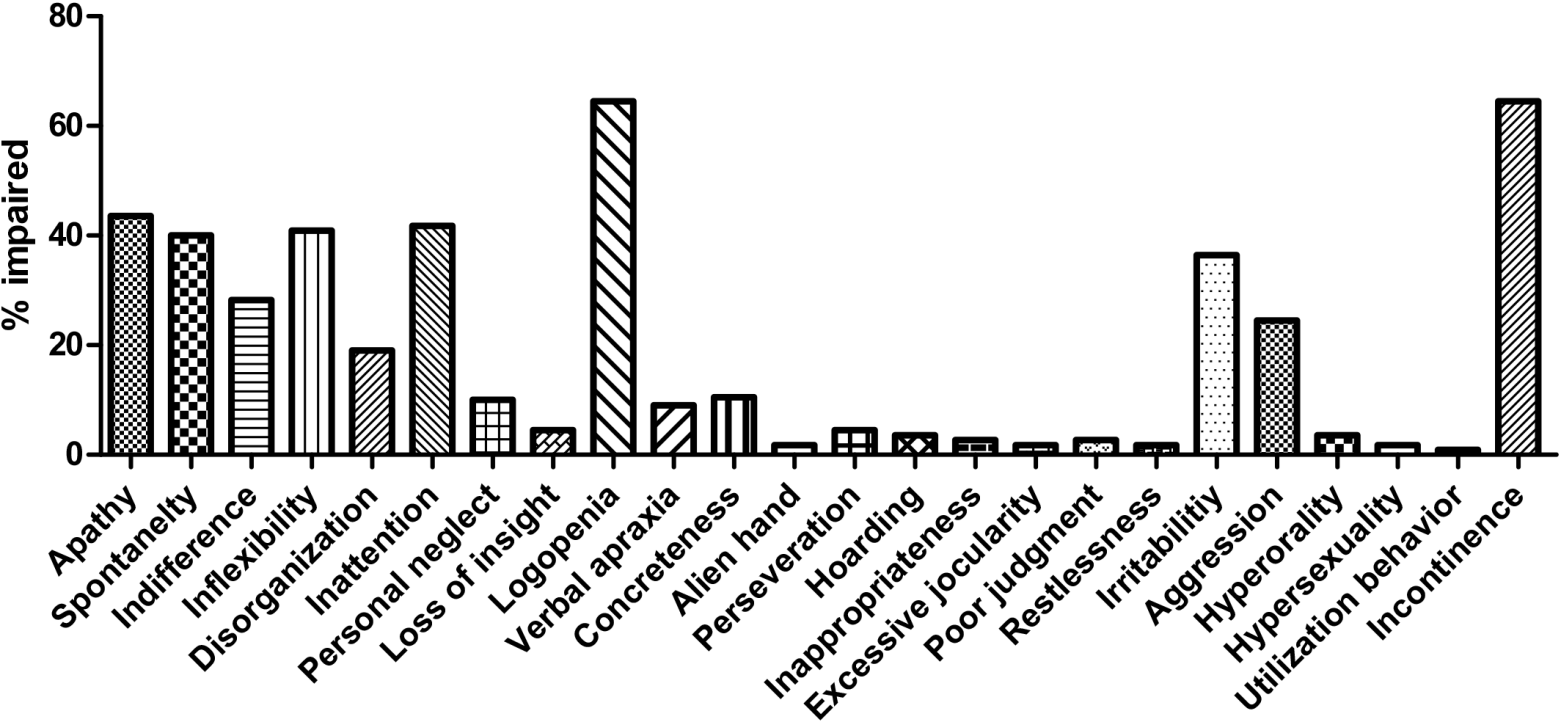
Histogram showing the proportion of MSA patients with each behavior on the FBI. The FBI traits are plotted in relation to the number of total subjects showing mild to severe behavioral changes.

The mean FBI score was 5.5 ± 4.5. Based on the FBI score, 22.7% patients showed mild frontal behavior changes (FBI score 1–3), 57.2% patients showed moderate frontal behavior changes (FBI score 4–15), and 2.7% patients showed severe behavior changes (FBI score>15). The most commonly impaired neurobehavioral domain was incontinence (71 patients, 64.5%), followed by logopenia (67 patients, 60.9%) and apathy (48 patients, 43.6%) (Fig 3). The UMSARS scores increased with increasing severity of frontal behavior changes (Table A in S1 File).

The results of Spearman’s correlations between MSA-related variables and the neurobehavioral test results are shown in Table 4. The total ACE-R score was correlated with the education level, and the UMSARS, FAB and FBI scores. The total FAB score was correlated with education level and the UMSARS, ACE-R and FBI scores. The total FBI score was correlated with the UMSARS, ACE-R and FAB scores.

**Table 4:** Spearman’s correlation coefficients between related clinical variables and total ACE-R, FAB, and FBI scores.

	ACER		FAB		FBI	
Variables	r_s_	*P-*value	r_s_	*P-*value	r_s_	*P-*value
Onset age	-0.045	0.641	-0.128	0.184	-0.031	0.748
Disease duration	-0.151	0.115	-0.117	0.223	0.063	0.512
Education	0.552	<0.0001	0.386	<0.0001	-0.054	0.574
UMSARS	-0.305	0.001	-0.302	0.001	0.236	0.013
ACE-R	-	-	0.668	<0.0001	-0.237	0.013
FAB	0.668	<0.0001	-	-	-0.244	0.01
FBI	-0.237	0.013	-0.244	0.01	-	-

MSA: Multiple system atrophy; FAB: Frontal Assessment Battery; FBI: Frontal Behavioral Inventory

UMSARS: Unified MSA Rating Scale; ACE-R: Addenbrooke's Cognitive Examination-Revised.

The binary logistic regression (Wald forward entry method) model revealed that education level < 9 years and UMSARS ≥ 40 were the potential determinants of abnormal ACE-R in MSA patients (Table 5), whereas in MSA-C patients,these two values were the potential determinants of abnormal FAB (Table 5).

**Table 5:** Regression analyses predicting the outcome, with adjustments for non-significant but clinically important covariates. Each row represents a separate regression analysis.

Outcome variables	Independent significant covariates	OR	Coefficient (95% CI)	*p*-value	Non-significant controlled covariates
ACE-R	Eudcation level	13.312	2.931–60.469	<0.0001	Onset age level, Gender, Sub-type, Disease duration level
	UMSARS level	2.444	1.002–5.962	0.049	
FAB	Subtype	4.326	1.631–11.477	0.003	Gender, Onset age level, Disease duration level
	Eudcation level	2.809	1060–7.444	0.038	
	UMSARS level	5.396	2.103–13.846	<0.0001	

MSA: Multiple system atrophy; FAB: Frontal Assessment Battery; FBI: Frontal Behavioral Inventory

UMSARS: Unified MSA Rating Scale; ACE-R: Addenbrooke's Cognitive Examination-Revise.

The MSA-C patients had lower UMSARS scores than MSA-P patients (P<0.05). After adjustment for the UMSARS score, MSA-C patients had lower total scores for FAB and the domain “conflicting instruction”, and a higher percentage of abnormal FAB than MSA-P patients (Table B in S1 File).

Compared with male MSA patients, the female patients had lower subtest scores for “similarity” of FAB (P<0.05). There were no significant differences between male and female patients in the subgroups regarding subtypes, years of education, disease duration, age of onset, and UMSARS, ACE-R, FBI and FAB scores (Table B in S1 File).

## Discussion

The present study is the first to evaluate global cognition and specific cognitive functions, including frontal lobe-related ability/executive function and behavior changes, in a large Chinese MSA patient population. Global cognitive deficits, frontal lobe dysfunction, and behavior changes were common in Chinese MSA patients. MSA patients with a low education level and severe motor deficit were more likely to have global cognitive deficits. Moreover, in the MSA-C subtype patients, low education level and severe motor deficit are the potential predictors of frontal lobe dysfunction.

Global cognitive deficits were commonly observed in the present study, showing that 32.7% and 20.9% of patients with MSA had global cognitive deficits based on ACE-R and MMSE, respectively, consistent with the findings of previous studies (ranged from 20% to 42.9%)[6,21]. These findings suggested that ACE-R is superior to MMSE for detecting cognitive deficits in MSA, particularly for detecting mild cognitive deficits, consistent with previous studies on other neurodegenerative diseases[15,16,18,19]. Considering the broader range of cognitive domains, ACE-R accurately and comprehensively reflects the cognitive profile of MSA patients and represents a more reliable bedside test to detect cognitive deficits than MMSE.

Wide ranges of cognitive domains defects, including visuospatial ability, verbal fluency, memory, language, and orientation/ attention, were observed in MSA patients, consistent with previous studies[6,7,21,30]. The high frequency of visuospatial disability has been confirmed in a series of studies[6,7]. The language and verbal fluency function, including spontaneous speech, syntax, repetition, and lexico-semantic function, have been confirmed in other studies on MSA patients[6,30]. These consistent findings suggest that Chinese patients with MSA have similar impairments in cognitive domains compared to non-Chinese MSA patients. Because patients with severe ataxia or parkinsonism symptoms were excluded, these findings suggest that cognitive dysfunction, particularly language and verbal fluency, is a feature of MSA, independent of motor deficits.

A recent meta-analysis on cognitive impairments in MSA patients showed that the main impaired domain of cognition was executive function[3]. The results of the present study showed that 34.5% of Chinese MSA patients had frontal lobe dysfunction, consistent with the findings of previous studies on Caucasian populations (ranging from 26.7% to 41%) [4,21,22]. In the present study, the most commonly affected domains in MSA patients were “inhibitory control” and “similarity”. The “similarity” function, representing the ability of abstracting processes, is heavily dependent on the activity in the dorsolateral prefrontal cortex (DLPFC)[31]. The function of “inhibitory control” is primarily associated with activity in the orbitofrontal cortex (OFC)[32]. Recent imaging[6–8,33] and pathological studies[10,34] have revealed frontal and temporal degeneration in patients with MSA. For example, an MRI voxel-based morphormetry study showed OFC atrophy in MSA patients[33], and[18F]fluorodeoxyglucose positron emission tomography studies showed decreased [18F]fluorodeoxyglucose uptake in the DLPFC of MSA patients[6,7]. These imaging and pathological studies supported the findings of the present study showing frontal lobe dysfunction in MSA patients.

The present study showed that 82.7% of patients with MSA had frontal behavior changes based on FBI; however, only 2.7% of patients had severe behavior changes, and most of the patients had mild and moderate behavior changes. Few studies have focused on frontal behavior changes in MSA patients. A small study including 39 MSA-P and 22 MSA-C Caucasian patients showed that the Neuropsychiatric Inventory (NPI) scores of both MSA-C and MSA-P patients were lower than those in the PD group, and there was no significant difference between the two subtypes[4]. Another small study including 10 MSA-C and 13 MSA-P Asian patients showed that MSA patients had higher NPI scores than healthy controls[8]. However, these two studies did not analyze the frequency of frontal behavior changes in MSA patients. The most commonly affected FBI domain was “incontinence”, reflecting the autonomic dysfunction of MSA patients. The other two most frequent neurobehavioral changes besides “incontinence”, i.e., logopenia and apathy, are negative symptoms. A study involving 34 MSA patients showed that these patients more frequently suffered from apathy than PD patients based on a non-motor symptoms scale, with a mean frequency of 65% in MSA patients, which is higher than the frequency of 43.6% obtained in the present study[35]. The difference in the frequency of apathy might reflect differences in the selected samples and scales. The positive correlation between frontal behavior changes and the UMSARS scores observed in the present study suggests that the frontal behavior changes reflect the disease progression in MSA patients. A previous study also showed that the NPI sub-items, such as agitation, disinhibition, aberrant motor behavior, and eating disorders, are correlated with the UMSARS score[4]. In the present study, we cannot completely exclude the impact of mood such as reactive depression, as we did not measure the mood using specific scales because of the rapid progression of MSA without any causal treatment option. Additionally, we selected the cut-off score used in previous studies[29,36] because we did not evaluate the FBI of healthy subjects. Therefore, these limitations should be considered when explaining MSA-specific findings with frontal behavioral changes.

The large sample size used in the present study also facilitated the examination of the natural history and clinical correlates between cognitive dysfunction and frontal lobe dysfunction in Chinese patients with MSA. Given the association between low education level and cognitive status in older populations[37], we matched the education years between patients and HCs. Therefore, the cognitive decline of patients with MSA was associated with the disease itself. The finding that MSA patients with low education levels and severe disease were more likely to have global cognitive deficits is consistent with the findings in PD[38]. Additionally, a previous study on MSA using the MMSE scale also showed that MSA patients with a lower education level were more likely to experience global cognitive impairment[21]. The findings of the present study were supported by the cognitive reserve hypothesis in AD, which proposes that higher education provides more efficient compensatory mechanisms against underlying pathology[39]. Another study on MSA showed that the global cognitive deficits were not associated with disease duration, consistent with the results of the present study[22].

Notably, the disease severity and MSA-C subtype are potential predictors of frontal lobe dysfunction. The relationship between disease severity and frontal lobe dysfunction has also been observed in previous studies, consistent with the findings of the present study[4,5]. Moreover, patients with MSA-C might be more likely to have frontal lobe dysfunction than those with MSA-P. A study from Taiwan showed that MSA-C patients performed worse than MSA-P patients in the memory score, Stroop test, and time to complete the trail-making test, which also supports the findings of the present study. The increased atrophy observed in MSA-C patients compared with MSA-P patients might explain the difference in the frontal lobe dysfunction between MSA-C and MSA-P patients[8]. Additionally, the disrupture of cerebella-thalamus-cortical loops might also play a role [40,41].

Additionally, the present study was one of a few studies to examine general cognitive function, frontal lobe dysfunction and behavior changes in two subtypes of MSA patients of different sexes. The results showed that the “similarity” score was lower in female than in male MSA patients. MSA was classified to be the “synucleinopathies” along with PD. Cognitive performance in PD is different between different sexes [42,43]. For example, Braak et al. reported that female PD patients frequently have more cognitive impairment (94.4%) than male PD patients (86.5%)[42]; Riedel et al. showed that female patients attained significantly worse score for Clock Drawing Test scores in a large and nationwide research study of PD patients[43]. The results of the present study suggested that sex might play a role in the frontal lobe dysfunction in MSA patients. However, the contribution of sex to the observed difference in the FAB of MSA patients remains unclear.

Some limitations cannot be ignored in the present study. First, the current study was a cross-sectional study and.we did not present the longitudinal data obtained on the frontal lobe dysfunction and behavior changes due to the limited time. However, we continue to explore the global cognition, frontal lobe dysfunction and behavior changes in MSA patients. Second, all patients in the present study were recruited from a single center in China. Therefore, it is necessary to conduct a multi-center prospective study on general cognition, frontal lobe function and behavior changes in MSA.

## Conclusion

Cognitive impairment is common in Chinese MSA patients. The findings of the present study strongly suggest that cognitive impairment should not be an exclusion criterion for the diagnosis of MSA in research and clinical trials. Low education level and disease severity are potential predictors of global cognitive deficits in MSA patients. Moreover, MSA-C subtype, disease severity, and low education level are potential predictors of frontal lobe function.

## Supporting Information

S1 File Table A. Demographic and clinical characteristics of MSA patients in terms of frontal behavior changes. Table B. Demographic and clinical measures according to clinical symptoms and sex. (DOCX)

## References

[1] Stefanova N, Bucke P, Duerr S, Wenning GK (2009) Multiple system atrophy: an update. Lancet Neurol 8: 1172–1178. 10.1016/S1474-4422(09)70288-1 19909915

[2] Gilman S, Wenning GK, Low PA, Brooks DJ, Mathias CJ, Trojanowski JQ, et al (2008) Second consensus statement on the diagnosis of multiple system atrophy. Neurology 71: 670–676. 10.1212/01.wnl.0000324625.00404.15 18725592 PMC2676993

[3] Stankovic I, Krismer F, Jesic A, Antonini A, Benke T, Brown RG, et al (2014) Cognitive impairment in multiple system atrophy: A position statement by the neuropsychology task force of the MDS multiple system atrophy (MODIMSA) study group. Mov Disord 29: 857–867. 10.1002/mds.25880 24753321 PMC4175376

[4] Siri C, Duerr S, Canesi M, Delazer M, Esselink R, Bloem BR, et al (2013) A cross-sectional multicenter study of cognitive and behavioural features in multiple system atrophy patients of the parkinsonian and cerebellar type. J Neural Transm 120: 613–618. 10.1007/s00702-013-0997-x 23462799

[5] Kim H-J, Jeon BS, Kim YE, Kim J-Y, Kim YK, Sohn C-H, et al (2013) Clinical and imaging characteristics of dementia in multiple system atrophy. Parkinsonism & Related Disorders 19: 617–621. 10.1016/j.parkreldis.2013.02.012 23529023

[6] Lyoo CH, Jeong Y, Ryu YH, Lee SY, Song TJ, Lee JH, et al (2008) Effects of disease duration on the clinical features and brain glucose metabolism in patients with mixed type multiple system atrophy. Brain 131: 438–446. 10.1093/brain/awm328 18178568

[7] Kawai Y, Suenaga M, Takeda A, Ito M, Watanabe H, Tanaka F, et al (2008) Cognitive impairments in multiple system atrophy: MSA-C vs MSA-P. Neurology 70: 1390–1396. 10.1212/01.wnl.0000310413.04462.6a 18413566

[8] Chang CC, Chang YY, Chang WN, Lee YC, Wang YL, Lui CC, et al (2009) Cognitive deficits in multiple system atrophy correlate with frontal atrophy and disease duration. Eur J Neurol 16: 1144–1150. 10.1111/j.1468-1331.2009.02661.x 19486137

[9] Wakabayashi K, Ikeuchi T, Ishikawa A, Takahashi H (1998) Multiple system atrophy with severe involvement of the motor cortical areas and cerebral white matter. Journal of the Neurological Sciences 156: 114–117. 9559999 10.1016/s0022-510x(98)00018-5

[10] Armstrong RA, Cairns NJ, Lantos PL (2007) A quantitative study of the pathological changes in white matter in multiple system atrophy. Neuropathology 27: 221–227. 17645235 10.1111/j.1440-1789.2007.00759.x

[11] Dos Santos Kawata KH, Hashimoto R, Nishio Y, Hayashi A, Ogawa N, Kanno S, et al (2012) A Validation Study of the Japanese Version of the Addenbrooke's Cognitive Examination-Revised. Dement Geriatr Cogn Dis Extra 2: 29–37. 10.1159/000336909 22619659 PMC3350351

[12] Kwak YT, Yang Y, Kim GW (2010) Korean Addenbrooke's Cognitive Examination Revised (K-ACER) for differential diagnosis of Alzheimer's disease and subcortical ischemic vascular dementia. Geriatr Gerontol Int 10: 295–301. 10.1111/j.1447-0594.2010.00624.x 20497240

[13] Fang R, Wang G, Huang Y, Zhuang JP, Tang HD, Wang Y, et al (2014) Validation of the Chinese version of Addenbrooke's cognitive examination-revised for screening mild Alzheimer's disease and mild cognitive impairment. Dement Geriatr Cogn Disord 37: 223–231. 10.1159/000353541 24193223

[14] Carvalho VA, Barbosa MT, Caramelli P (2010) Brazilian version of the Addenbrooke Cognitive Examination-revised in the diagnosis of mild Alzheimer disease. Cogn Behav Neurol 23: 8–13. 10.1097/WNN.0b013e3181c5e2e5 20299857

[15] Kaszas B, Kovacs N, Balas I, Kallai J, Aschermann Z, Kerekes Z, et al (2012) Sensitivity and specificity of Addenbrooke's Cognitive Examination, Mattis Dementia Rating Scale, Frontal Assessment Battery and Mini Mental State Examination for diagnosing dementia in Parkinson's disease. Parkinsonism Relat Disord 18: 553–556. 10.1016/j.parkreldis.2012.02.010 22405839

[16] Bak TH, Rogers TT, Crawford LM, Hearn VC, Mathuranath PS, Hodges JR (2005) Cognitive bedside assessment in atypical parkinsonian syndromes. J Neurol Neurosurg Psychiatry 76: 420–422. 15716539 10.1136/jnnp.2003.029595 PMC1739531

[17] Begeti F, Tan AY, Cummins GA, Collins LM, Guzman NV, Mason SL, et al (2013) The Addenbrooke's Cognitive Examination-Revised accurately detects cognitive decline in Huntington's disease. J Neurol 260: 2777–2785. 10.1007/s00415-013-7061-5 23922130

[18] Law E, Connelly PJ, Randall E, McNeill C, Fox HC, Parra MA, et al (2013) Does the Addenbrooke's Cognitive Examination-revised add to the Mini-Mental State Examination in established Alzheimer disease? Results from a national dementia research register. Int J Geriatr Psychiatry 28: 351–355. 10.1002/gps.3828 22556006

[19] Wei Q, Chen X, Zheng Z, Huang R, Guo X, Cao B, et al (2015) Screening for cognitive impairment in a Chinese ALS population. Amyotroph Lateral Scler Frontotemporal Degener 16: 40–45. 10.3109/21678421.2014.966311 25309978

[20] Lima CF, Meireles LP, Fonseca R, Castro SL, Garrett C (2008) The Frontal Assessment Battery (FAB) in Parkinson's disease and correlations with formal measures of executive functioning. J Neurol 255: 1756–1761. 10.1007/s00415-008-0024-6 18821046

[21] Brown RG, Lacomblez L, Landwehrmeyer BG, Bak T, Uttner I, Dubois B, et al (2010) Cognitive impairment in patients with multiple system atrophy and progressive supranuclear palsy. Brain 133: 2382–2393. 10.1093/brain/awq158 20576697

[22] Kawamura K, Shimohata T, Nakayama H, Tomita M, Ozawa T, Nishizawa M (2010) Factors influencing the cognitive function in patients with multiple system atrophy. Mov Disord 25: 2891–2892. 10.1002/mds.23260 20925069

[23] Kertesz A, Davidson W, Fox H (1997) Frontal behavioral inventory: diagnostic criteria for frontal lobe dementia. Can J Neurol Sci 24: 29–36. 9043744 10.1017/s0317167100021053

[24] Geser F, Wenning GK, Seppi K, Stampfer-Kountchev M, Scherfler C, Sawires M, et al (2006) Progression of multiple system atrophy (MSA): a prospective natural history study by the European MSA Study Group (EMSA SG). Mov Disord 21: 179–186. 16161136 10.1002/mds.20678

[25] Yabe I, Soma H, Takei A, Fujiki N, Yanagihara T, Sasaki H (2006) MSA-C is the predominant clinical phenotype of MSA in Japan: analysis of 142 patients with probable MSA. J Neurol Sci 249: 115–121. 16828805 10.1016/j.jns.2006.05.064

[26] Wenning GK, Tison F, Seppi K, Sampaio C, Diem A, Yekhlef F, et al (2004) Development and validation of the Unified Multiple System Atrophy Rating Scale (UMSARS). Mov Disord 19: 1391–1402. 15452868 10.1002/mds.20255

[27] Litvan I, Aarsland D, Adler CH, Goldman JG, Kulisevsky J, Mollenhauer B, et al (2011) MDS Task Force on mild cognitive impairment in Parkinson's disease: critical review of PD-MCI. Mov Disord 26: 1814–1824. 10.1002/mds.23823 21661055 PMC3181006

[28] Szeto JY, Mowszowski L, Gilat M, Walton CC, Naismith SL, Lewis SJ (2015) Assessing the utility of the Movement Disorder Society Task Force Level 1 diagnostic criteria for mild cognitive impairment in Parkinson's disease. Parkinsonism Relat Disord 21: 31–35. 10.1016/j.parkreldis.2014.10.020 25465744

[29] Josephs KA, Whitwell JL, Eggers SD, Senjem ML, Jack CR Jr (2011) Gray matter correlates of behavioral severity in progressive supranuclear palsy. Mov Disord 26: 493–498. 10.1002/mds.23471 21462261

[30] Kao AW, Racine CA, Quitania LC, Kramer JH, Christine CW, Miller BL (2009) Cognitive and neuropsychiatric profile of the synucleinopathies: Parkinson disease, dementia with Lewy bodies, and multiple system atrophy. Alzheimer Dis Assoc Disord 23: 365–370. 10.1097/WAD.0b013e3181b5065d 19935145 PMC2886667

[31] MacPherson SE, Phillips LH, Della Sala S (2002) Age, executive function, and social decision making: a dorsolateral prefrontal theory of cognitive aging. Psychol Aging 17: 598–609. 12507357

[32] Langenecker SA, Nielson KA, Rao SM (2004) fMRI of healthy older adults during Stroop interference. Neuroimage 21: 192–200. 14741656 10.1016/j.neuroimage.2003.08.027

[33] Brenneis C, Egger K, Scherfler C, Seppi K, Schocke M, Poewe W, et al (2007) Progression of brain atrophy in multiple system atrophy. Journal of Neurology 254: 191–196. 17334661 10.1007/s00415-006-0325-6

[34] Konagaya M, Sakai M, Matsuoka Y, Konagaya Y, Hashizume Y (1999) Multiple system atrophy with remarkable frontal lobe atrophy. Acta Neuropathologica 97: 423–428. 10208284 10.1007/s004010051008

[35] Colosimo C, Morgante L, Antonini A, Barone P, Avarello TP, Bottacchi E, et al (2010) Non-motor symptoms in atypical and secondary parkinsonism: the PRIAMO study. Journal of Neurology 257: 5–14. 10.1007/s00415-009-5255-7 19669613

[36] Wei Q, Chen X, Zheng Z, Huang R, Guo X, Cao B, et al (2014) Frontal lobe function and behavioral changes in amyotrophic lateral sclerosis: a study from Southwest China. J Neurol 261: 2393–2400. 10.1007/s00415-014-7508-3 25249295

[37] Evans DA, Beckett LA, Albert MS, Hebert LE, Scherr PA, Funkenstein HH, et al (1993) Level of education and change in cognitive function in a community population of older persons. Ann Epidemiol 3: 71–77. 8287159 10.1016/1047-2797(93)90012-s

[38] Komadina NC, Terpening Z, Huang Y, Halliday GM, Naismith SL, Lewis SJ (2011) Utility and limitations of Addenbrooke's Cognitive Examination-Revised for detecting mild cognitive impairment in Parkinson's disease. Dement Geriatr Cogn Disord 31: 349–357. 10.1159/000328165 21613789

[39] Kemppainen NM, Aalto S, Karrasch M, Nagren K, Savisto N, Oikonen V, et al (2008) Cognitive reserve hypothesis: Pittsburgh Compound B and fluorodeoxyglucose positron emission tomography in relation to education in mild Alzheimer's disease. Ann Neurol 63: 112–118. 18023012 10.1002/ana.21212

[40] Houck BD, Person AL (2014) Cerebellar loops: a review of the nucleocortical pathway. Cerebellum 13: 378–385. 10.1007/s12311-013-0543-2 24362758 PMC4207368

[41] Salmi J, Pallesen KJ, Neuvonen T, Brattico E, Korvenoja A, Salonen O, et al (2010) Cognitive and motor loops of the human cerebro-cerebellar system. J Cogn Neurosci 22: 2663–2676. 10.1162/jocn.2009.21382 19925191

[42] Braak H, Rub U, Jansen Steur EN, Del Tredici K, de Vos RA (2005) Cognitive status correlates with neuropathologic stage in Parkinson disease. Neurology 64: 1404–1410. 15851731 10.1212/01.WNL.0000158422.41380.82

[43] Ghosh BC, Carpenter RH, Rowe JB (2013) A longitudinal study of motor, oculomotor and cognitive function in progressive supranuclear palsy. PLoS One 8: e74486 10.1371/journal.pone.0074486 24058574 PMC3769232

