# Supplementary material for: The Global Cognition, Frontal Lobe Dysfunction and Behavior Changes in Chinese Patients with Multiple System Atrophy
Source: PLoS One. 2015 Oct 2;10(10):e0139773. doi: 10.1371/journal.pone.0139773 (PMC4591982; doi:10.1371/journal.pone.0139773)
Supplement: S1 File — (DOCX) [file pone.0139773.s001.docx]

Table A in Supplement Table. Demographic and clinical characteristics of MSA patients in terms of frontal behavior change.

| Variants | FBI score  (0)  n=19 | FBI score  (1-3)  n=25 | FBI score  (4-15)  n=63 | FBI score  (>15)  n=3 | P value |
| --- | --- | --- | --- | --- | --- |
| Subtype  MSA-C/MSA-P | 13/6 | 12/13 | 39/24 | 3/0 | 0.246 |
| Men/women | 12/7 | 15/10 | 33/30 | 2/1 | 0.795 |
| Age of onset (years) | 57.7±10.0 | 56.0±8.1 | 56.6±10.0 | 57.4±6.1 | 0.942 |
| Years of education | 10.2±4.4 | 8.5±3.4 | 9.7±3.6 | 7.0±1.7 | 0.266 |
| Disease duration | 2.3±1.2 | 2.6±1.5 | 2.9±1.5 | 2.2±1.3 | 0.446 |
| UMSARS | 31.4±10.8 | 39.1±14.5 | 41.1±11.7 | 38.3±3.1 | 0.030 |
| FAB score | 15.1±3.9 | 14.8±2.7 | 14.1±3.0 | 11.7±5.0 | 0.270 |
| Similarity | 3.0(2.0, 3.0) | 3.0(2.0, 3.0) | 2.0(2.0, 3.0) | 3.0(2.0, 3.0) | 0.707 |
| Lexical fluency | 3.0(2.0, 3.0) | 3.0(2.0, 3.0) | 3.0(2.0, 3.0) | 3.0(1.0, 3.0) | 0.693 |
| Motor series | 3.0(1.0, 3.0) | 3.0(2.0, 3.0) | 3.0(2.0, 3.0) | 2.0(2.0, 3.0) | 0.989 |
| Conflicting instruction | 3.0(2.0, 3.0) | 3.0(2.0, 3.0) | 3.0(1.0, 3.0) | 1.0(0.0, 3.0) | 0.126 |
| Go-no-go task | 3.0(2.0, 3.0) | 2.0(1.0, 3.0) | 2.0(1.0, 3.0) | 0.0(0.0, 3.0) | 0.245 |
| Prehension behavior | 3.0(3.0, 3.0) | 3.0(3.0, 3.0) | 3.0(3.0, 3.0) | 0.0(0.0, 3.0) | 0.095 |
| FAB abnormal | 5(26.3%) | 8(32%) | 23(36.5%) | 2(66.7%) | 0.561 |
| ACE-R score | 79.7±12.2 | 72.8±14.5 | 73.1±12.6 | 61.7±19.4 | 0.090 |
| Orientation/attention score | 16.7±1.5 | 16.6±1.8 | 16.6±1.7 | 15.7±2.1 | 0807 |
| Memory score | 21.8±3.0 | 20.0±5.3 | 19.8±4.2 | 16.3±6.7 | 0.154 |
| Verbal fluency score | 8.9±2.8 | 8.3±2.2 | 7.2±3.0 | 4.0±3.0 | 0.013 |
| Language score | 20.0±5.3 | 16.1±5.4 | 17.9±4.9 | 15.7±7.2 | 0.084 |
| Visuospatial ability score | 12.3±3.9 | 11.8±3.7 | 11.6±3.2 | 10.0±5.0 | 0.715 |
| ACE-R abnormal | 4(21.1%) | 9(36%) | 21(33.4%) | 2(66.7%) | 0.411 |
| Orientation/attention abnormal | 2(10.5%) | 3(12%) | 6(9.6%) | 1(33.4%) | 0.635 |
| Memory abnormal | 3(15.8%) | 8(32%) | 14(23.8%) | 2(66.7%) | 0.204 |
| Verbal fluency abnormal | 2(10.5%) | 4(20%) | 21(33.4%) | 2(66.7%) | 0.048 |
| Language abnormal | 3(15.8%) | 9(36%) | 15(23.8%) | 2(66.7%) | 0.171 |
| Visuospatial ability abnormal | 5(26.3%) | 7(28%) | 16(25.4%) | 1(33.4%) | 0.987 |

Table B in Supplement Table Demographic and clinical measures according to clinical symptoms and sex

| Variants | MSA  (n=110) |  |  | clinical symptoms | | |  | sex | | |  |
| --- | --- | --- | --- | --- | --- | --- | --- | --- | --- | --- | --- |
|  |  |  |  | MSA-C  (n=67) | MSA-P  (n=43) | P-value |  | males  (n=62) | females  (n=48) | P-value |  |
| Men/women | 62/48 |  |  | 38/29 | 24/19 | 0.926 |  | - | - | - |  |
| Age of onset (years) | 56.7±9.4 |  |  | 55.3±9.6 | 58.8±8.8 | 0.054 |  | 55.7±9.7 | 58.0±9.0 | 0.224 |  |
| Years of education | 9.4±3.7 |  |  | 9.16±3.6 | 9.9±3.9 | 0.322 |  | 9.5±3.7 | 9.4±3.7 | 0.953 |  |
| Disease duration | 2.7±1.5 |  |  | 2.6±1.5 | 2.8±1.4 | 0.590 |  | 2.8±1.5 | 2.6±1.4 | 0.477 |  |
| UMSARS | 38.9±12.5 |  |  | 37.0±11.5 | 42.0±13.6 | 0.040 |  | 37.8±13.2 | 41.3±11.6 | 0.300 |  |
| ACE-R score | 73.9±13.4 |  |  | 73.4±13.6 | 74.7±13.2 | 0.624 |  | 75.6±12.2 | 71.6±14.6 | 0.117 |  |
| Orientation/attention score | 16.6±1.7 |  |  | 16.6±1.6 | 16.61.9 | 0.927 |  | 16.8±1.5 | 16.3±1.9 | 0.298 |  |
| Memory score | 20.1±4.4 |  |  | 20.0±4.8 | 20.3±3.9 | 0.723 |  | 20.5±4.1 | 19.5±4.8 | 0.359 |  |
| Verbal fluency score | 7.7±2.9 |  |  | 7.3±3.0 | 8.2±2.9 | 0.143 |  | 8.2±2.7 | 7.0±3.2 | 0.167 |  |
| Language score | 17.8±5.2 |  |  | 17.6±5.3 | 18.0±5.1 | 0.733 |  | 18.1±5.5 | 17.4±4.9 | 0.472 |  |
| Visuospatial ability score | 11.7±3.5 |  |  | 11.8±3.5 | 11.6±3.5 | 0.795 |  | 12.0±3.4 | 11.3±3.5 | 0.280 |  |
| ACE-R abnormal | 36(32.7%） |  |  | 23（34.3%） | 13(30.2%） | 0.444 |  | 17(27.4%) | 19(39.6%) | 0.178 |  |
| Orientation/attention abnormal | 12(10.9%) |  |  | 6(9.0%) | 6(14.0%) | 0.659 |  | 4(6.4%) | 8(16.7%) | 0.088 |  |
| Memory abnormal | 27(24.5%) |  |  | 18(26.9%) | 9(21.0%) | 0.219 |  | 11(17.7%) | 16(33.3%) | 0.060 |  |
| Verbal fluency abnormal | 29(26.3%) |  |  | 19(28.4$) | 10(23.2%) | 0.359 |  | 15(24.2%) | 14(29.2%) | 0.557 |  |
| Language abnormal | 29(26.3%) |  |  | 21(31.3%) | 8(18.65) | 0.070 |  | 17(27.4%) | 12(25%) | 0.775 |  |
| Visuospatial ability abnormal | 29(26.3%) |  |  | 18(26.9%) | 11(25.6%) | 0.394 |  | 14(22.6%) | 15(31.3%) | 0.306 |  |
| FAB score | 14.4±3.2 |  |  | 14.0±3.2 | 15.0±3.2 | 0.044 |  | 14.6±3.0 | 14.13.4 | 0.572 |  |
| Similarity | 2.4±0.7 |  |  | 2.0(2.0, 3.0) | 3.0(2.0, 3.0) | 0.224 |  | 3.0(2.0, 3.0) | 2.0(2.0, 3.0) | 0.037 |  |
| Lexical fluency | 2.6±0.6 |  |  | 3.0(2.0, 3.0) | 3.0(2.0, 3.0) | 0.765 |  | 3.0(2.0, 3.0) | 3.0(2.0, 3.0) | 0.784 |  |
| Motor series | 2.3±0.9 |  |  | 3.0(1.0, 3.0) | 3.0(2.0, 3.0) | 0.225 |  | 3.0(1.0, 3.0) | 3.0(2.0, 3.0) | 0.882 |  |
| Conflicting instruction | 2.3±1.0 |  |  | 2.0(1.0, 3.0) | 3.0(2.0, 3.0) | 0.032 |  | 3.0(1.75, 3.0) | 3.0(2.0, 3.0) | 0.594 |  |
| Go-no-go task | 1.9±1.0 |  |  | 2.0(1.0, 3.0) | 2.0(1.0, 3.0) | 0.271 |  | 2.0(1.0, 3.0) | 3.0(1.25, 3.0) | 0.912 |  |
| Prehension behavior | 2.9±0.5 |  |  | 3.0(3.0, 3.0) | 3.0(3.0, 3.0) | 0.364 |  | 3.0(3.0, 3.0) | 2.0(1.0, 3.0) | 0.244 |  |
| FAB abnormal | 38(34.5%) |  |  | 29(43.2%) | 9(20.9%) | 0.001 |  | 21(33.9%) | 17(35.4%) | 0.866 |  |
| FBI score | 5.5±4.5 |  |  | 5.8±5.0 | 5.2±3.8 | 0.502 |  | 5.1±4.7 | 6.0±4.3 | 0.175 |  |
| FBI positive symptoms | 1.3±1.7 |  |  | 0.0(0.0, 2.0) | 0.0(0.0, 2.0) | 0.697 |  | 0.5(0.0, 3.0) | 0.0(0.0, 2.0) | 0.531 |  |
| FBI negative symptoms | 4.2±3.7 |  |  | 3.0(1.0, 6.0) | 3.0(2.0, 6.0) | 0.525 |  | 3.0(1.0, 5.0) | 4.5(2.0, 7.0) | 0.095 |  |
| FBI abnormal | 91(82.7%) |  |  | 54（80.6%） | 37（86%） | 0.842 |  | 50(80.6%) | 41(97.6%) | 0.511 |  |
| mild behavior changes | 25(22.7%) |  |  | 13(19.4%) | 12(27.9%) | 0.380 |  | 15(24.2%) | 10(20.8%) | 0.355 |  |
| moderate behavior changes | 63(57.2%) |  |  | 39(61.9%) | 24(55.8%) | 0.455 |  | 33(53.2%) | 30(62.5%) | 0.329 |  |
| severe behavior changes | 3(2.7%) |  |  | 3(4.4%) | 0(0%) | 0.390 |  | 2(3.2%) | 1(2.0%) | 1.000 |  |

Abbreviations: MSA: Multiple system atrophy; FAB: Frontal Assessment Battery; FBI : Frontal Behavioral Inventory; UMSARS: Unified MSA Rating Scale; ACE-R : Addenbrooke's Cognitive Examination-Revised;
